# Etymologia: Ignatzschineria

**DOI:** 10.3201/eid2110.ET2110

**Published:** 2015-10

**Keywords:** etymologia, Ignatzschineria, bacteria, gram negative, nonmotile rods, flies, Ignatz Rudolph Schiner

## *Ignatzschineria* [ig-natʺshi-nerʹe-ə]

A genus of aerobic, gram-negative, nonmotile rods, *Ignatzschineria* was first isolated from flies of the family *Sarcophagidae* (from the Greek *sarco* [“flesh”] + *phage* [“eating”]) by Erika Tóth et al. in 2001. Tóth named the genus after Austrian entomologist Ignaz Rudolph Schiner (1813–1873), who first described the fly *Wohlfahrtia magnifica*. In 2007, Tóth discovered that *Schineria* had already been used for genus of tachina flies and proposed the replacement genus name *Ignatzschineria*.
